# Gardening in Childcare Centers: A Randomized Controlled Trial Examining the Effects of a Garden Intervention on Physical Activity among Children Aged 3–5 Years in North Carolina

**DOI:** 10.3390/ijerph20115939

**Published:** 2023-05-24

**Authors:** Nancy M. Wells, Nilda Graciela Cosco, Derek Hales, Muntazar Monsur, Robin C. Moore

**Affiliations:** 1 Department of Human Centered Design, College of Human Ecology, Cornell University, 1300F MVR Hall, Ithaca, NY 14853, USA; 2 Department of Landscape Architecture and Environmental Planning, College of Design, North Carolina State University, 50 Pullen Road, Raleigh, NC 27695, USA; 3 Center for Health Promotion and Disease Prevention, University of North Carolina at Chapel Hill, 1700 Martin L. King Jr. Blvd., CB 7426, Chapel Hill, NC 27599, USA; 4 Department of Landscape Architecture, Davis College of Agricultural Sciences and Natural Resources, Texas Tech University, Lubbock, TX 79409, USA

**Keywords:** childcare, children, gardening, randomized controlled trial, physical activity, accelerometry

## Abstract

This study examined the effects of a childcare gardening intervention on children’s physical activity (PA). Eligible childcare centers were randomly assigned to: (1) garden intervention (*n* = 5; year 1); (2) waitlist control (*n* = 5; control year 1, intervention year 2); or (3) control (*n* = 5; year 2 only) groups. Across the two-year study, PA was measured for 3 days at four data collection periods using Actigraph GT3X+ accelerometers. The intervention comprised 6 raised fruit and vegetable garden beds and a gardening guide with age-appropriate learning activities. The sample included a total of 321 3–5-year-olds enrolled in childcare centers in Wake County, North Carolina, with *n* = 293 possessing PA data for at least one time point. The analyses employed repeated measures linear mixed models (SAS v 9.4 PROC MIXED), accounting for clustering of the children within the center and relevant covariates (e.g., cohort, weather, outside days, accelerometer wear). A significant intervention effect was found for MVPA (*p* < 0.0001) and SED minutes (*p* = 0.0004), with children at intervention centers acquiring approximately 6 min more MVPA and 14 min less sedentary time each day. The effects were moderated by sex and age, with a stronger impact for boys and the youngest children. The results suggest that childcare gardening has potential as a PA intervention.

## 1. Introduction

Most preschool children in the United States are not meeting the recommended levels of physical activity (PA) [1,2,3]. Among children aged 3–5 years, approximately 42% of boys and 60% of girls do not meet the recommended daily 3 h of PA [4]. Childhood inactivity is associated with a variety of adverse outcomes, including poor cognition, lower academic performance, delayed motor skill development, worse cardiorespiratory health, and obesity [2,5,6,7,8,9,10,11,12,13,14,15]. Moreover, insufficient PA early in life can set a child on a trajectory toward lifelong physical inactivity, overweight, and associated adverse health outcomes across the life course [15,16], making early childhood a critical period for PA intervention.

Serving 12.5 million children in the U.S. [17,18], childcare centers or early childhood environments (ECE) are increasingly recognized as a compelling context for health promotion and obesity prevention [19,20,21]. The policies, practices, and environmental features of ECEs significantly influence the PA of young children [22,23,24,25]; accounting for as much as 43.3% and 30.9% of the variance in daily moderate to vigorous PA (MVPA) and vigorous PA (VPA), respectively [26]. However, children are sedentary during most of the time spent in childcare [26,27].

Fruit and vegetable (FV) gardens have the potential to affect children’s health behaviors, including dietary intake [28,29] and PA [30,31], in addition to providing opportunities for pedagogical instruction in areas such as science, math, and language arts [32,33]. Gardening offers children a variety of activities involving a range of PA intensities from low (e.g., transplanting, mixing growing medium, harvesting), to moderate (e.g., weeding, mulching), to high intensity (e.g., digging, raking) [34,35]. However, research has seldom examined gardening in the childcare setting. The few studies of gardening and PA among youth have tended to focus on school-age children (i.e., ~8–12 year-olds) [36,37,38], with little attention paid to young children within ECEs [39,40]. This is a critical gap, given the importance of the childcare setting and the urgency for early intervention.

This study aims to fill gaps in the literature by examining what effect a childcare gardening intervention has on the PA of young children within an under-resourced community. This work is theoretically grounded in Barker’s concept of a behavior setting—a physical area in which certain types of behaviors are supported [41]; in Bronfenbrenner’s bioecological model that suggests that the various contexts or “microsystems” of daily life (e.g., home, childcare, school, work) shape human development and behavior [42,43]; and in the life course perspective, which underscores the critical role of early experiences in the formation of lifelong habits and health outcomes [44,45]. We hypothesize that in comparison to those in the control group(s), children who participate in the garden intervention will be (a) more physically active and (b) less sedentary, as measured by accelerometry while in the ECE. We also examine the moderating effects of sex and age to consider whether the intervention exerts different impacts on boys vs girls, as well as on children at different ages.

## 2. Method

### 2.1. Research Design

This study employed a waitlist-control group, RCT, to assess the impact of the Preventing Obesity by Design (POD) Gardening intervention [46] on PA among 3–5-year-old children enrolled in 15 ECEs in Wake County, North Carolina. The larger study also examined the effects of the intervention on children’s fruit and vegetable identification, liking, and consumption [28]. The protocol is detailed elsewhere [47] and described briefly here. 

The 15 ECEs were randomly assigned to three groups. The 15 ECEs were randomly assigned to one of three groups. Assignment was stratified by the percent of children at an ECE receiving outside funding to pay cost of attending (subsidization level) in order to balance this factor across groups. Group 1, “intervention”, received the intervention in year 1 (5 centers); Group 2, “waitlist control”, or “delayed intervention”, served as the control in year 1 and received the intervention in year 2 (5 centers); and Group 3, “no-intervention control, joined the study in year 2 as a control group and did not receive the garden intervention (5 centers). As an incentive, Group 3 received the garden installation and training resources upon the completion of the study. Baseline data were collected in spring, year 1. Group 1 (intervention) then received the garden intervention in the summer of year 1. Follow-up data collection for Groups 1 and 2 was completed in early fall, following the intervention. In spring of year 2, the procedures were repeated, collecting baseline data from Groups 2 and 3. Group 2, the waitlist control ECEs, then received the garden intervention in summer, year 2. In fall of year 2, follow-up data were collected for Groups 2 and 3. Thus, Groups 1 and 3 each have one pre-intervention measurement and Group 2 has three measurements prior to the intervention. Group 2′s multiple pre-tests enhance the research design by allowing us to rule out alternative explanations (threats to internal validity) [48]. The study is registered with ClinicalTrials.gov, #NCT04864574. The North Carolina State University Institutional Review Board (IRB) approved the research design and methods (protocol approval #5908).

### 2.2. Setting: Childcare Centers + Center Recruitment

A total of 310 licensed Wake County ECEs were identified in collaboration with Wake County Smart Start. Of those, 23 ECEs met the initial qualification criteria and were invited to complete an online application to verify eligibility (e.g., having a 4- or 5-star rating from state licensing authority; serving a majority of children eligible for a childcare subsidy program; containing at least two preschool classrooms; not currently having a garden). All identified ECEs completed the application, and 15 ECEs were randomly assigned to Groups 1, 2, and 3. The remaining 8 ECEs served as a back-up pool. See Cosco et al. [28,47] for complete eligibility criteria.

### 2.3. Participants

Participants were children aged 3–5 who attended the 15 selected ECEs. A total of 543 children were eligible from the pool of the 15 selected ECEs. Of those, 321 received parental consent to participate and provided some PA or demographic data. Of those consented, 293 (91%) had PA data at one or more time points. The sample and data collection flow diagram are available in Figure S1.

### 2.4. Constructs and Measures

#### 2.4.1. Independent Variable: The Garden Intervention 

**Figure 1:**
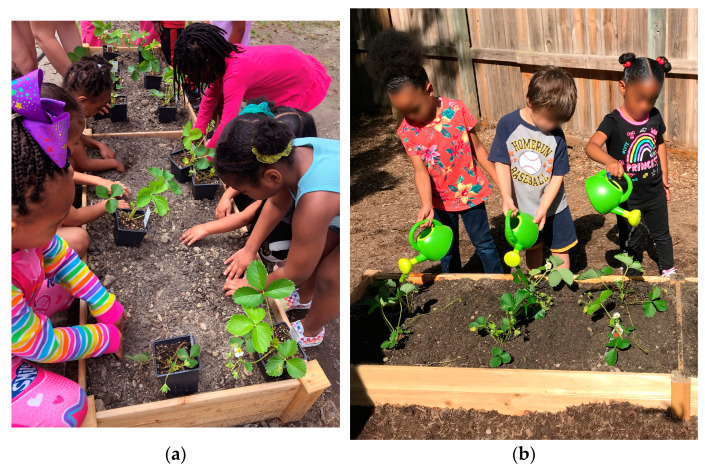
Children gardening: (**a**) planting strawberries; (**b**) watering strawberries.

The intervention comprised six raised garden beds, specified FV plantings, a seasonal planting plan, classroom activities for garden engagement, and weekly on-site technical assistance from research staff with gardening expertise. Six vegetables (cucumbers, green beans, green peppers, tomatoes, yellow squash, and zucchini) and five fruits (blackberries, blueberries, cantaloupe, strawberries, and watermelon) were included (Figure 1). “The Garden Activity Guide” was provided to each participating classroom. The guide contained 12 age-appropriate gardening activities, including children’s literature suggestions, and was organized using three themes: Preparing, Caring, and Harvesting/Eating (e.g., examining seeds, preparing garden beds, watering, weeding, etc.) [28,47]. Teachers typically led the activities, which occurred outdoors, 3–4 times per week for 30 min. 

#### 2.4.2. Dependent Variable: Physical Activity 

The PA of the children was measured using ActiGraph GT3X+ accelerometers [49]. Children’s accelerometry data are highly correlated with energy expenditure, oxygen consumption, heart rate, and treadmill speed [50,51]. MVPA and Sedentary minutes per day served as the two primary PA indicators for analysis. Secondary PA indicators included light, moderate, and vigorous intensity, vector magnitude per minute (“total counts” per minute), and steps per day. All intensity outcomes are presented as minutes per day and percentage of the day (computed as: 100*[intensity minutes/wear minutes]).

### 2.5. Procedure

Data Collection. Children wore Actigraph GT3X+ accelerometers during childcare hours for three consecutive days at each data collection timepoint. The accelerometers were attached to the waist of each child with a static nylon belt (aligned with the right hip) by trained research staff on the morning of each data collection day. The accelerometers were labelled to ensured that children wore the same one each day. On each data collection day, research staff or trained teachers logged the on and off times for each participant and recorded the start and end of nap time. After data collection was complete, raw data (collected at 40 Hz) were downloaded and converted to 5-s epoch-level files using ActiLife software version 6.13 [49]. Non-wear, wear, and sleep (nap) were assessed using the Choi algorithm, data collection logs, custom algorithms, and visual inspection of the data [52]. Days with at least 270 min of waking wear were considered complete (~75% of the ~360-min childcare day). For each day meeting the wear minute criteria, cut-points were used to determine minutes of sedentary (<8.3 counts/5 s), light (8.4–191 counts/5 s), moderate (192–334 counts/5 s), and vigorous (≥335 counts/5 s) activity [53]. Intensity minute outcomes were also converted to % time variables (e.g., % sedentary = 100 ∗ [minutes sedentary/total wear minutes]). Data summarizing MVPA and Sedentary time were primary to the assessment of the intervention. Participants with at least one day meeting the wear criteria were kept in the final dataset. In our sample, 11% of children had 1 day, 28%—2 days, and 60%—3 days of data. The percentage of children with only 1 day of good wear did not significantly differ by time point (pre/post) or group (intervention/control), ranging from 10.4% to 12.7%. Because the focus of the analysis (next section) was children clustered within centers, having additional children and days to represent the overall activity of the center was beneficial. An additional analysis (unpublished) including only children with 2+ days produced results similar to those presented. Comparison of completers (children with baseline (BL) and follow-up (FU) data) to those with only BL PA outcomes revealed a slight difference in #days of accelerometer wear, 2.6 days for completers vs 2.4, and a higher level of missing (29% vs 4%) for child race/ethnicity in the BL-only group. None of the primary PA outcomes differed significantly in this comparison (see Table S1). Processing and cleaning were conducted in SAS (v9.4), as were all statistical analyses.

### 2.6. Statistical Analysis

Chi-square tests and simple general linear models were used for frequency and mean comparisons between groups (INT vs. CON) and cohorts (year 1 vs. year 2) at baseline. Primary analyses of the intervention effects were conducted using repeated measures linear mixed models with maximum likelihood estimation, accounting for clustering of children within the center (SAS PROC MIXED). Comparison of covariance structures using Akaike (AIC) and Bayesian information criteria values suggested very little difference for model estimation between covariance types; in the final models, autoregressive (AR1) structures were utilized. For each PA outcome, three models were fit. Models were progressive, with each including additional covariates. Model 1 included accelerometer wear time; Model 2 added the number of days with no outside time (0–2), the number of days with rain (0–2), and the cohort (year 1 or 2); and Model 3 added the child’s age at baseline (years) and the child’s sex (girl or boy). Note that for outcomes that included wear time in the calculation (e.g., % time MVPA), the models did not include wear time. The adjusted least squares means and standard errors for Model 3 are also presented. The percent change (100*[[MEANfu-MEANbl]/MEANbl]) from baseline and the effect size ([INT mean change—CON mean change]/pooled baseline SD) were also computed for each PA outcome.

Exploratory analyses were conducted to examine the moderating effects of child sex and child age at baseline for MVPA and Sedentary time. For each, a 3-way (GROUP × TIME × [AGE or SEX] [AGE or SEX]) interaction term was added to Model 3. For child age, the model was run with age as a continuous variable, as well as with age as a 3-level categorical variable. To examine the stability of our results, sensitivity analyses were conducted by fitting models in two sub-samples: (1) removing children who had abnormal days (days with rain or no outside time) and children with very high or low activity estimates (*n* available = 327), and (2) keeping only completers, or children with BL and FU measurements (*n* available = 331). For the 5 ECE with measures at all 4 timepoints (Y1-Control; Y2-Intervention), an additional interrupted time series model (SAS PROC Model [%itsa by Caswell [54,55,56]]) was fitted to the data. This was used to compare the behavior change trajectories (slopes) pre- and post-intervention. All analyses were conducted on both primary (MVPA and Sedentary) and secondary PA outcomes.

## 3. Results

At baseline (see Table 1), children (3.9 ± 0.53 years; 51% girls; 58.6 ± 28.9 BMI%) accumulated 34.8 ± 12.6 min of MVPA and 273 ± 37 min of sedentary time each day. Accelerometer wear days, child sex and race distribution, BMI percentile (i.e., a child’s height and weight relative to other children of the same age and sex) [57], and PA outcomes were similar across groups. While age, number of days with no outside time, and number of days with rain were different across both group and cohorts. Cohort differences were also noted for several PA outcomes, with children in cohort 2 (i.e., year 2) starting out as slightly less active compared to cohort 1 (i.e., year 1). The final analysis sample included 500 observations from the 293 children with PA data; 47.1% of children had data at only one timepoint. Intraclass correlation coefficients for the clustering of children within the center ranged from 0.19 to 0.24 for PA outcomes and increased slightly at follow-up (range 0.25 to 0.29).

The group means, change, and results from models used to test intervention effects can be found in Table 2. The GROUP × TIME intervention effects for the models including all covariates (Model 3) were statistically significant and in the expected direction for both MVPA and Sedentary outcomes. While all covariates remained in the final models, only wear time, sex, and number of days with no outside time remained statistically significant (*p* < 0.05; see Table S2). The adjusted models suggest that the intervention group achieved about 6 more minutes of MVPA (~20%) and about 14 minutes less sedentary time (~5%) following the intervention period compared to the control group. The intervention effects were also statistically significant for nearly all the secondary PA outcomes, with differences between the intervention and control groups of 7% (% time light; n.s. *p* = 0.057) to 24% (vigorous minutes; *p* = 0.0003). The results of the sensitivity analyses support the primary findings, with slightly decreased effects in the “no abnormal days” analysis and slightly stronger effects for MVPA for completers only.

**Figure 2:**
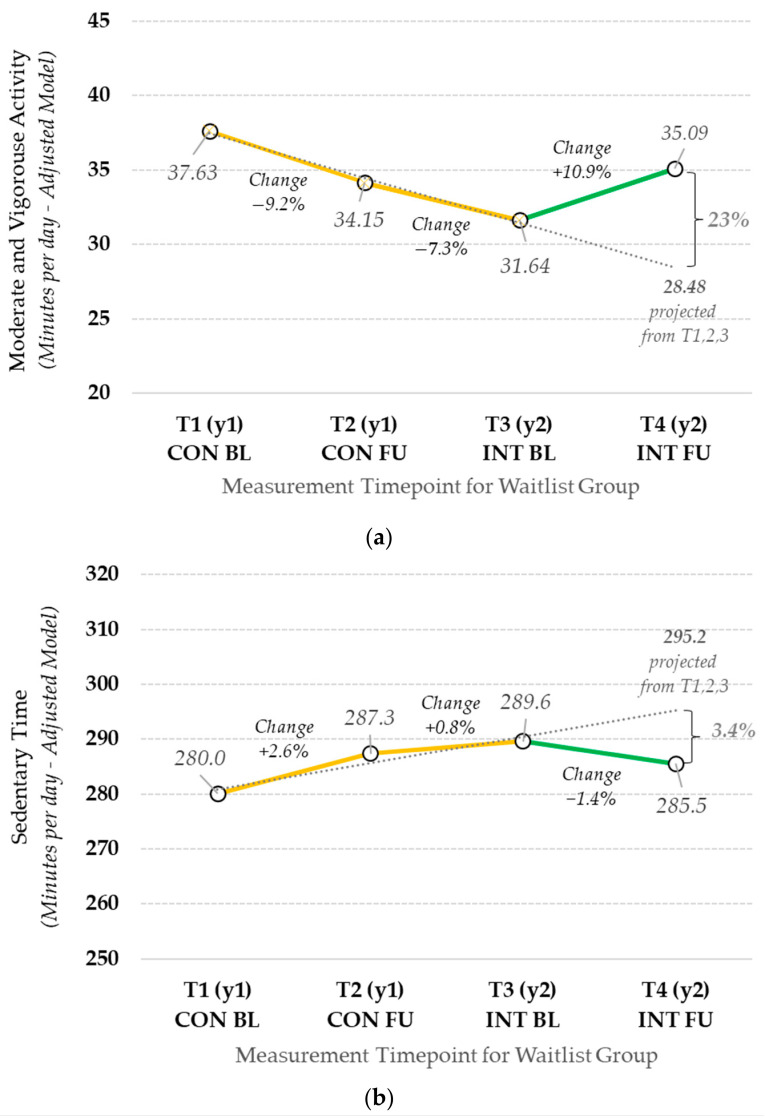
Change in outcome variables from T1 to T4: (**a**) change in MVPA per day for the five waitlist control centers; (**b**) change in sedentary minutes per day for the five waitlist control centers—year 1, control (CON); year 2, intervention (INT)—at baseline (BL) and follow-up (FU)).

The change in MVPA and Sedentary minutes per day for the 5 waitlist centers (*n* = 144 children) over the 4 measurement timepoints is shown in Figure 2. The results of the interrupted time series model, with standard errors adjusted for autocorrelation, suggest that the trajectory for MVPA before and after the intervention was significantly different (*p* = 0.022); however, changes for sedentary time were not. During the pre-intervention control period (year 1), children saw decreases in MVPA (T1→T2: −9.2%; T2→T3: −7.3%) and slight increases in sedentary time ((T1→T2: 2.6%; T2→T3: 0.8%). During the intervention period, MVPA increase by about 11%, which was similar to the change seen in the primary and sensitivity analyses for the full sample. Sedentary time decreased slightly during the intervention year (−1.4%; change not statistically significant).

**Figure 3:**
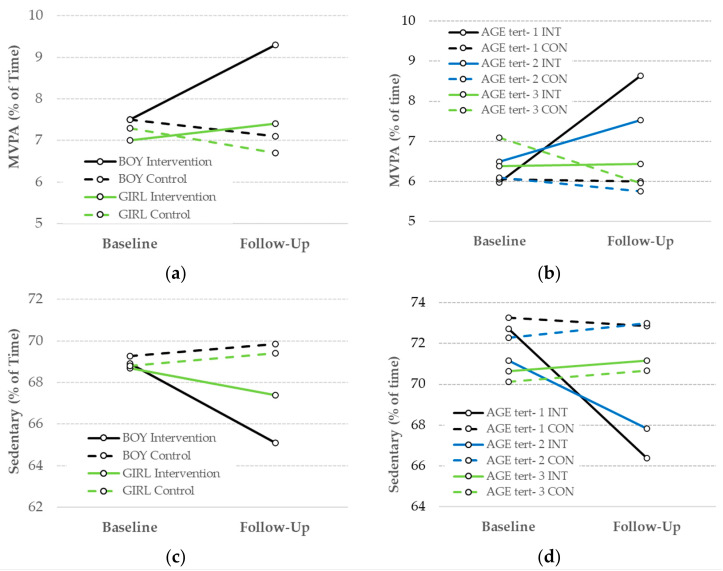
Age and sex moderate the effect of the garden intervention on MVPA and sedentary time: (**a**) sex moderates the effect of intervention on MVPA; (**b**) age moderates the effect of intervention on MVPA; (**c**) sex moderates the effect of the intervention on sedentary time; (**d**) age moderates the effect of the intervention on sedentary time.

The results suggest that the moderating effect of child sex was only statistically significant for MVPA (*p* = 0.021; Sedentary *p* = 0.210). Complete model results can be found in Table S2. Figure 3 shows the adjusted baseline and follow-up MVPA and sedentary time for the GROUP × TIME × Moderator effects. For boys, the intervention produced a significantly larger change in MVPA (28.4% increase) than for girls (12.9% increase). For sedentary time, the interaction was not statistically significant, but boys in the intervention centers showed double the decrease (−5.5 % vs. −2.8%) in sedentary time compared to girls. The results also suggest that the intervention effects were moderated by age. For both MVPA (*p* = 0.015) and sedentary time (*p* = 0.009), the interactions were significant, with the impact strongest for the youngest tertile (mean age = 3.3 year), positive but slightly less for middle tertile (mean age = 3.8 years), and minimal to zero for the oldest tertile (mean age = 4.5 years).

## 4. Discussion

Our study, the first RCT to examine the effects of a childcare-based gardening intervention on the PA of young children, indicates that gardening increases PA and reduces sedentary behavior within the ECE. These findings are consistent with prior research conducted with older children [36,38] and extend our understanding of gardening’s effects on PA to early childhood. The examination of the garden as an additional behavior setting [41] within the outdoor learning environment (OLE) of ECEs builds upon research documenting that the adjacency—or connectedness—of behavior settings within the childcare OLE is positively associated with PA [24]. Our findings are also consistent with Cosco’s [58] findings suggesting that PA among preschool children could be affected by a diversity of settings and the mix of natural and manufactured elements within the OLE.

While the effects were positive for all children, sex moderated the impact of the intervention on MVPA. The effects of the intervention were considerably stronger for boys than for girls. Given that boys are typically more physically active than girls in early childhood [59,60] and beyond [61,62] (and indeed, in this study, boys were more active than girls at baseline), the stronger intervention effect for boys is consistent with the phenomenon known as the Matthew Effect. This effect refers to an intervention’s amplification of pre-existing advantages [63] and has been documented across varied domains, including reading [64], academic achievement [65], medicine [63], and PA interventions [66,67,68]. Secondly, age moderated the effects of the intervention on both MVPA and sedentary time. The youngest children were the most strongly affected by the intervention, closely followed by the middle age group. However, the MVPA and sedentary time of the oldest group (mean 4.5 years) was scarcely impacted. This finding is consistent with prior evidence suggesting that “the earlier the better” is the most effective time for interventions targeting health behaviors such as PA [15,69]. This study makes an important contribution as the first to identify differential effects of a gardening intervention on PA by sex and age. Differential impacts of PA interventions are common, yet under-reported [66,70], and urgently merit attention if we are to understand issues of PA equity and disparity across population subgroups [70].

### 4.1. Strengths and Limitations

This RCT has many strengths. The robust research design employs random assignment, uses a waitlist control group, takes place over two years, and has a substantial sample size, all of which help to ensure strong internal validity. The inclusion of a waitlist control group strengthens the study by allowing for the examination of outcomes at three timepoints prior to the intervention. The trends evident in this group across 18 months (i.e., declining MVPA and slighting increasing sedentary time) prior to the intervention, followed by a directional shift after the intervention, help us to rule-out a variety of threats to internal validity (i.e., alternative explanations), such as local history, selection by maturation, and differential regression to the mean [48]. Regarding construct validity, accelerometry is considered the gold standard for operationalizing PA, as it provides an objective measure that avoids the myriad challenges of self-report measures. The study also fills several important gaps in the literature. Chief among the gaps addressed is the focus on the understudied childcare context, on early childhood, and on a low-income community where children are particularly at risk for physical inactivity, sedentary behavior, poor fitness, and associated health effects [71,72].

This study is not without limitation. While the study of childcare centers in low-income communities is critical, such a focus presents a variety of practical research challenges. Enrollment within the childcare center tends to be unstable, teacher retention is often low, and leadership changes are not infrequent, all of which create additional challenges to navigate, including attrition. The differential drop in sample size for the intervention (31%) vs. control (12%) was not ideal, but the similarity in results for the completers only (sensitivity analysis) and full sample analysis do support our findings and alleviate worries about the impact of sample loss on the results. Unfortunately, the data documenting the reasons for children missing follow-up were incomplete, with about 60% of children missing this information; 35% had left the center, and 4% had moved to a different class (not in the study). This study did not include a process analysis to determine whether, and at which centers, the intervention was delivered “as intended.” Moreover, despite the merits of accelerometry, some nuances of PA (i.e., weight-bearing tasks) and activity context are not captured and may require direct observation (e.g., Myers + Wells [73]). PA was not measured at home. It is possible that the effects of the intervention “carried over” to the home, or conversely, the children may have become less active at home as compensation for the additional MVPA at the ECE. Finally, this study was intentionally conducted in an under-resourced area of North Carolina, which may impact the external validity or generalizability to other communities.

### 4.2. Implications

This research has compelling implications for both policy and practice. Our findings suggest that a gardening intervention within childcare will indeed nudge PA upward and reduce sedentary time. With intervention work, it is important to consider the potential accumulated effects of small changes over time. A 5–10% daily increase can seem trivial (e.g., 3 min/day), but if it persists, can quickly add up. For example, our results suggest that in an average childcare day (~8 h), a gardening intervention might add 6 MVPA minutes and reduce sedentary time by 16 min/day compared to the rates in centers with no gardens. Over the course of the 4-month intervention period, children attending centers with gardens may accumulate 8 additional hours of MVPA (6 min/day ∗ 80 days) and 21+ fewer hours of sedentary time. While the duration of outdoor time was not measured, changes in MVPA and sedentary time are likely related to increased time outdoors [74], indicating that gardening interventions could be promoted as a means of helping ECEs meet or exceed outdoor play best practice recommendations and state-level requirements [75,76,77,78,79,80,81].

The findings regarding the roles of age and sex as moderators of the relationship between gardening and children’s MVPA and sedentary time are important for both clinical implementation and research design/testing. If educators or extension agents are aware that programs function better for a given group, modifications can be implemented to improve the impact for all. Or, programs can be targeted toward those for whom they are most effective, maximizing efficiencies. In future work, we plan to examine the consistency of these effects and their potential causes in more detail. Teacher behavior and differences in activity choices may play a role in these moderating effects. The addition of direct observation and staff reporting may help identify unexplored influences on children’s movement behaviors.

### 4.3. Future Research

There are many avenues for future research under the umbrella of the influence of gardening on children’s PA. Here, we consider three key themes: mediating mechanisms, moderators (interactions), and the life course perspective.

Mediating mechanism(s) illuminate how and why gardening affects PA. A compelling candidate as a mediator of the garden–PA relationship is motivation. In their seminal work on motivation, Yarrow and colleagues [82] considered the roles of variety, complexity, and responsiveness as characteristics of environments that encourage children’s activity. FV gardens encompass these attributes by offering seasonal changes, a variety of plant types (fruits, vegetables, herbs, trees), sensory stimulation (textures, flavors, aromas), changes in dimension (slow/rapid growth), and a rewarding response to garden care activities (e.g., watering, weeding). A clearer understanding of how motivation may operate as an explanatory mechanism linking garden characteristics to PA would contribute to our theoretical understanding and guide in plant selection and the designing of childcare garden interventions. Another plausible mediator is time outdoors. While the impact of the gardening intervention on PA was significant, it is unclear if the PA increase was due to garden-specific movement or to an increase in time spent outside, which is a consistent, reliable predictor of PA among children [83,84]. An important next step will be to identify the types of activities, environments, and behaviors that change during a garden intervention, which may illuminate the mediating mechanisms.

Second, moderators are a critical topic for research. Differential effects of PA interventions across sub-groups—as we document in this study—may contribute to inequities, not only in PA, but ultimately, in health outcomes [70]. Given that the effects on MVPA were greater for boys than for girls, this issue is particularly salient with respect to gender-based PA disparities. Young girls are less likely than young boys to achieve recommended levels of PA, and gender disparities in PA expand throughout childhood and into adolescence [1,85]. Future research ought to identify ways to amplify the effects of PA interventions for girls and mitigate the exacerbation of the gender gap [70]. Targeted, rather than universalized, interventions merit further study [63]. Perhaps the effects would be greater in a gender-segregated garden intervention or with the addition of interventions in line with research indicating that teachers’ modelling of PA has a significant effect [86]. Modelling—particularly by female teachers—may be a strategy to increase the effects on girls’ PA.

Another compelling, though challenging, direction for future research is to expand beyond the longitudinal scope of this 2-year study to complete a “life course” study, implemented across decades. Such an investigation would endeavor to assess whether gardening experienced in early childhood sparks a turning point in PA and health trajectories [44], leading to positive long term health outcomes, such as longevity and the absence of disease. Parallel research might consider the influence of childhood gardening on outcomes such as time in nature throughout the life course [87], as well as pro-environmental attitudes and behaviors [88,89]. Studies might consider at what critical period(s) a garden intervention is most impactful. While our findings suggest that the youngest children experienced the greatest impact, it is possible that other critical periods exist across the life course [90].

## 5. Conclusions

This RCT examining childcare center gardening in an under-resourced area of North Carolina suggests that gardening early in life may be one strategy to bolster PA and reduce sedentary time among young children. By placing greater emphasis on gardening-based education as a childcare best practice, state-level requirements and quality standards could bolster children’s health and wellness across a range of domains (e.g., activity, nutrition, science learning, social interaction) with the incorporation of just one activity.

## Figures and Tables

**Table 1:** Comparison of sample at baseline.

		Intervention vs. Control at Baseline			Cohort 1 vs. 2 at Baseline
		INT (*n* = 140)	CON (*n* = 140)		
Environment		Percent	Percent	*p*-value ^a^	*p*-value ^a^
Number of Days with no outside time during measurement	*0 days*	92.9	100.0	0.001	0.001
	*1 day*	7.1	0.0		
	*2 days*	0.0	0.0		
Number of Days with rain during measurement	*0 days*	65.7	80.7	<0.001	<0.001
	*1 day*	10.7	19.3		
	*2 days*	23.6	0.0		
**Child**					
Race	*White*	35.7	30.0	0.732	0.175
	*Black*	41.4	40.7		
	*Hispanic*	3.6	5.7		
	*Other*	8.6	10.0		
	*Missing*	10.7	13.6		
Sex	*Girl*	50.7	50.7	0.598	0.836
	*Boy*	48.6	47.1		
	*Missing*	0.7	2.1		
		**MN (SD)**	**MN (SD)**	***p*** **-value ^b^**	***p*-value ^b^**
Age	*Years*	4.01 (0.58)	3.76 (0.44)	<0.001	<0.001
BMI percentile	*%-tile*	58.79 (29.6)	58.37 (28.3)	0.918	0.938
**Physical Activity**					
Wear Days	*per week*	2.59 (0.65)	2.48 (0.70)	0.186	0.996
Wear Hours	*per day*	6.91 (0.83)	7.06 (0.76)	0.104	<0.001
Vector Magnitude	*Per min*	1256 (306)	1266 (319)	0.780	0.007
Vertical Axis Cnts	*Per min*	563 (158)	556 (159)	0.733	0.008
Moderate and Vigorous	*min/day*	34.33 (12.8)	35.17 (12.5)	0.582	<0.001
	*% of Day*	8.17 (2.7)	8.27 (2.8)	0.752	0.003
Sedentary	*min/day*	270 (35)	275 (40)	0.337	0.320
	*% of Day*	65.7 (7.2)	65.0 (7.1)	0.459	0.003

NOTE: MN = mean; SD = standard deviation; CON = control; INT= intervention. ^a^ *p*-value for frequency comparison from Chi-square model. ^b^ *p*-value for mean comparison from general linear model.

**Table 2:** Change in physical activity outcomes.

			Unadjusted Means				Intervention Effects					Adjusted Means				
Physical Activity Outcomes			Baseline		Follow-Up		Change		Group × Time Interact			Baseline		Follow-Up		Change
			Mean	SD	Mean	SD	%	ES	M1 sig	M2 sig	M3 sig	MN	SE	MN	SE	%
Primary																
Moderate and Vigorous	Minutes per day	int	35.17	12.45	35.57	14.29	1.1	0.17	0.0038	0.0001	0.0001	30.8	2.2	34.8	2.2	12.8
		con	34.33	12.77	32.60	11.41	−5.0					31.6	2.4	29.3	2.3	−7.1
	% Day	int	8.27	2.83	8.54	3.25	3.3	0.25	0.0199	0.0002	0.0003	7.0	0.5	8.0	0.5	13.8
		con	8.17	2.74	7.74	2.63	−5.3					7.1	0.6	6.7	0.6	−5.9
Sedentary	Minutes per day	int	274.72	39.53	266.42	37.25	−3.0	−0.44	0.0357	0.0003	0.0009	286.2	5.3	276.4	5.2	−3.4
		con	270.43	34.96	278.55	39.78	3.0					286.4	5.7	290.0	5.4	1.3
	% Day	int	65.02	7.06	64.60	7.94	−0.6	−0.17	0.2407	0.0023	0.0058	69.8	1.3	67.4	1.3	−3.5
		con	65.65	7.15	66.42	7.09	1.2					70.4	1.4	70.7	1.3	0.5
**Secondary**																
Vector Magnitude	Per minute	int	1256	306	1312	360	4.5	0.30	0.0160	0.0002	0.0005	1090	60	1214	60	11.4
		con	1266	319	1228	322	−3.0					1107	64	1081	62	−2.3
Steps	Per day	int	3543	931	3567	1047	0.7	0.19	0.0044	0.0001	0.0002	3071	161	3372	159	9.8
		con	3446	1057	3286	911	−4.7					3046	172	2891	165	−5.1
Light Intensity	Minutes per day	int	113.79	26.15	112.14	26.58	−1.5	−0.03	0.1627	0.0052	0.0124	101.7	3.8	107.7	3.8	5.9
		con	109.60	29.03	108.79	25.07	−0.7					100.9	4.1	99.4	3.9	−1.4
	% Day	int	26.70	5.11	26.86	5.45	0.6	0.10	0.6314	0.0257	0.0569	23.2	0.9	24.6	0.9	6.3
		con	26.18	5.24	25.84	5.18	−1.3					22.6	1.0	22.7	1.0	0.3
Moderate Intensity	Minutes per day	int	21.52	7.31	20.81	7.9	−3.3	0.10	0.0150	0.0004	0.0006	19.1	1.3	20.5	1.3	7.5
		con	20.75	7.65	19.27	6.61	−7.1					19.0	1.4	17.2	1.3	−9.5
	% Day	int	5.05	1.61	4.99	1.81	−1.2	0.19	0.0716	0.0011	0.0017	4.3	0.3	4.7	0.3	8.7
		con	4.93	1.60	4.57	1.49	−7.3					4.2	0.3	3.9	0.3	−8.1
Vigorous Intensity	Minutes per day	int	13.65	5.74	14.76	6.79	8.1	0.23	0.0037	0.0002	0.0003	11.9	1.1	14.3	1.0	20.7
		con	13.59	5.86	13.34	5.35	−1.8					12.6	1.1	12.2	1.1	−3.3
	% Day	int	3.23	1.35	3.54	1.54	9.6	0.28	0.0118	0.0004	0.0006	2.8	0.3	3.3	0.2	21.1
		con	3.24	1.33	3.17	1.27	−2.2					2.9	0.3	2.8	0.3	−2.5

NOTE: MN = mean; SD = standard deviation; SE = standard error; ES = effect size (Cohen D); sig = *p*-value; CON = control; INT = intervention; BL = baseline; FU = follow-up; ICC = intraclass correlation coefficient (clustering). Sample size: CON BL *n* = 140; FU *n* = 123; INT BL *n* = 140; FU *n* = 97. Model 1 (M1): clustering and wear hours per day; Model 2 (M2): clustering, wear, cohort, rain, outside days; Model 3 (M3): clustering, wear, cohort, rain days, outside days, age at baseline, sex.

## Data Availability

The data presented in this study are available upon request from the 2nd author (Cosco). The data are not publicly available due to the study’s focus on young children.

## Supplementary Materials

The following supporting information can be downloaded at: https://www.mdpi.com/article/10.3390/ijerph20115939/s1, Figure S1: CONSORT flow diagram: sample and data collection flow; Table S1: Comparison of baseline information for children with baseline and follow-up physical activity data vs. children with only baseline physical activity outcomes; Table S2: Interaction effect results for models examining differences in intervention effects for boys vs. girls (sex) and age group (3 levels).
