# Supplementary material for: Gardening in Childcare Centers: A Randomized Controlled Trial Examining the Effects of a Garden Intervention on Physical Activity among Children Aged 3–5 Years in North Carolina
Source: Int J Environ Res Public Health. 2023 May 24;20(11):5939. doi: 10.3390/ijerph20115939 (PMC10252818; doi:10.3390/ijerph20115939)
Supplement: Supplementary file 1 [file ijerph-20-05939-s001.zip › ijerph-2259672-supplementary.pdf]

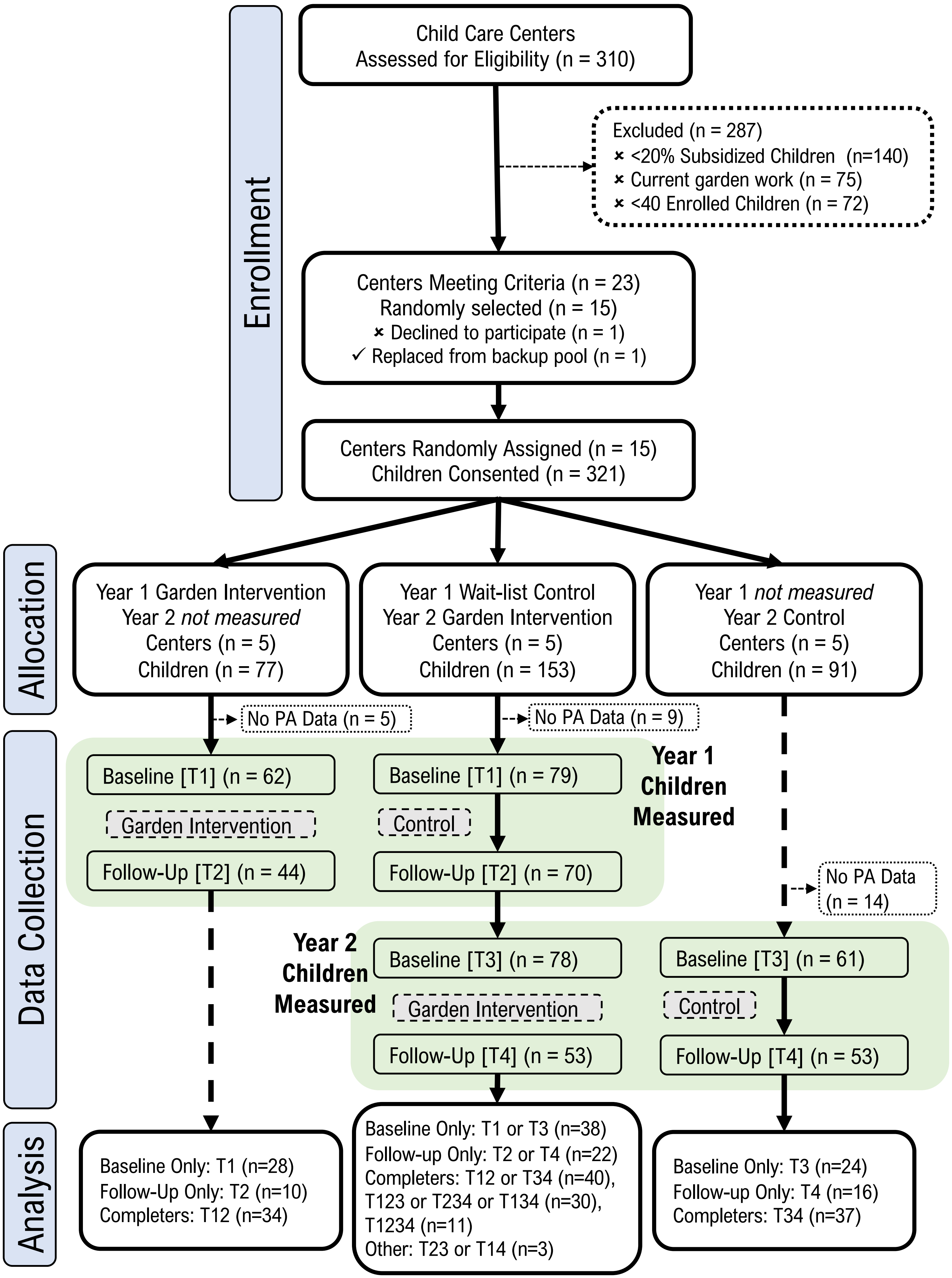
Enrollment
Child Care Centers
Assessed for Eligibility (n = 310)
Excluded (n = 287)
× <20% Subsidized Children (n=140)
× Current garden work (n = 75)
× <40 Enrolled Children (n = 72)
Centers Meeting Criteria (n = 23)
Randomly selected (n = 15)
× Declined to participate (n = 1)
✓ Replaced from backup pool (n = 1)
Centers Randomly Assigned (n = 15)
Children Consented (n = 321)
Allocation
Year 1 Garden Intervention
Year 2 not measured
Centers (n = 5)
Children (n = 77)
Year 1 Wait-list Control
Year 2 Garden Intervention
Centers (n = 5)
Children (n = 153)
Year 1 not measured
Year 2 Control
Centers (n = 5)
Children (n = 91)
Data Collection
No PA Data (n = 5)
No PA Data (n = 9)
Year 1 Children Measured
Baseline [T1] (n = 62)
Garden Intervention
Follow-Up [T2] (n = 44)
Baseline [T1] (n = 79)
Control
Follow-Up [T2] (n = 70)
No PA Data (n = 14)
Year 2 Children Measured
Baseline [T3] (n = 78)
Garden Intervention
Follow-Up [T4] (n = 53)
Baseline [T3] (n = 61)
Control
Follow-Up [T4] (n = 53)
Analysis
Baseline Only: T1 (n=28)
Follow-Up Only: T2 (n=10)
Completers: T12 (n=34)
Baseline Only: T1 or T3 (n=38)
Follow-up Only: T2 or T4 (n=22)
Completers: T12 or T34 (n=40),
T123 or T234 or T134 (n=30),
T1234 (n=11)
Other: T23 or T14 (n=3)
Baseline Only: T3 (n=24)
Follow-up Only: T4 (n=16)
Completers: T34 (n=37)

Table S1: Comparison of baseline information for children with baseline (BL) and follow-up (FU) physical activity data v. children with only BL physical activity outcomes

		Children with (BL and FU) Vs. (BL Only) at Baseline		
		BL and FU (n=190)	BL only (n=90)	
		Percent	Percent	p-value[a]
Number of Days no outside time during measurement	*0 days*	96.3	96.7	*0.883*
	1 day	3.7	3.3	
	2 days	0	0	
Number of Days with rain during measurement	*0 days*	72.1	75.6	*0.155*
	1 day	13.7	17.8	
	2 days	14.2	6.7	
**Child**				
Race	*White*	36.3	25.6	*< 0.001*
	Black	42.6	37.8	
	Hispanic	5.8	2.2	
	Other	11.1	5.6	
	Missing	4.2	28.9	
Sex	*Girl*	55.3	41.1	*0.076*
	Boy	43.7	56.7	
	Missing	1.1	2.2	
		MN (SD)	MN (SD)	p-value[b]
Age	*Years*	3.89 (0.48)	3.87 (0.63)	*0.714*
BMI percentile	*%-tile*	57.85 (29.1)	60.21 (28.7)	*0.594*
**Physical Activity**				
Wear Days	*per week*	2.59 (0.65)	2.41 (0.72)	*0.039*
Wear Hours	*per day*	6.98 (0.78)	6.99 (0.83)	*0.895*
Vector Magnitude	*Per min*	1275 (324)	1231 (285)	*0.268*
Moderate and Vigorous	*min/day*	34.6 (12.5)	35.0 (12.8)	*0.813*
	% of Day	8.21 (2.8)	8.25 (2.7)	*0.906*
Sedentary	*min/day*	270 (37)	278 (38)	*0.108*
	% of Day	64.8 (7.3)	66.5 (6.5)	*0.060*

NOTE: MN = Mean, SD = Standard Deviation, CON=control; INT= Intervention

[a] *p-value for frequency comparison from Chi-square model*

[b] *p-value for mean comparison from General linear model*

Table S2: Means and model summary for tests of GROUP x TIME x SEX interaction.

				UnAdjusted Means									Adjusted Means				
				Baseline			Follow-up			Change		Interaction	Baseline		Follow-up		
		SEX	Group	N	MEAN	SD	N	MEAN	SD	% Change	Effect Size	p-value	MEAN	SE	MEAN	SE	% Change
Moderate and Vigorous	Minutes per day	BOY	INT	68	36.19	12.93	41	40.64	13.20	12.3	0.41	*0.019*	32.13	2.36	39.45	2.48	22.8
		BOY	CON	66	33.65	13.23	57	32.77	12.02	-2.6			32.13	2.54	30.33	2.44	-5.6
		GIRL	INT	71	34.28	12.06	53	31.60	14.30	-7.8	-0.01		29.83	2.37	31.21	2.34	4.6
		GIRL	CON	71	35.02	12.65	64	32.45	11.11	-7.3			31.34	2.49	28.73	2.43	-8.3
	% of Day	BOY	INT	68	8.55	2.91	41	9.85	2.96	15.2	0.60	*0.021*	7.34	0.57	9.11	0.60	24.2
		BOY	CON	66	8.14	2.87	57	7.71	2.77	-5.3			7.21	0.60	6.94	0.59	-3.8
		GIRL	INT	71	8.03	2.76	53	7.47	3.18	-7.0	-0.04		6.81	0.57	7.17	0.57	5.3
		GIRL	CON	71	8.21	2.68	64	7.77	2.57	-5.4			7.06	0.60	6.51	0.58	-7.8
Sedentary Time	Minutes per day	BOY	INT	68	274.87	42.57	41	255.83	31.40	-6.9	-0.95	*0.155*	286.46	5.68	270.14	6.01	-5.7
		BOY	CON	66	268.77	31.28	57	284.73	38.18	5.9			287.29	6.16	290.39	5.90	1.1
		GIRL	INT	71	274.33	36.92	53	276.51	38.19	0.8	0.02		285.45	5.72	280.56	5.63	-1.7
		GIRL	CON	71	271.42	38.15	64	272.79	41.07	0.5			284.68	6.03	288.90	5.87	1.5
	% of Day	BOY	INT	68	65.31	7.18	41	62.96	6.98	-3.6	-0.45	*0.210*	69.91	1.40	66.05	1.49	-5.5
		BOY	CON	66	66.44	7.03	57	67.28	7.31	1.3			70.68	1.50	70.68	1.46	0.0
		GIRL	INT	71	64.72	7.03	53	66.08	8.34	2.1	0.09		69.60	1.42	68.29	1.40	-1.9
		GIRL	CON	71	64.88	7.24	64	65.59	6.94	1.1			69.88	1.49	70.50	1.45	0.9

Means and model summary for tests of GROUP x TIME x AGE (baseline) interaction

				UnAdjusted Means									Adjusted Means				
				Baseline			Follow-up			Change		Interaction	Baseline		Follow-up		
		AGE	Group	N	MEAN	SD	N	MEAN	SD	% Change	Effect Size	p-value	MEAN	SE	MEAN	SE	% Change
Moderate and Vigorous	Minutes per Day	Tert1	INT	35	34.17	12.00	19	34.86	12.87	2.0	0.15	*Tert - 0.024*	26.2	3.2	37.1	3.2	41.2
			CON	56	35.77	11.62	49	34.71	10.76	-3.0		*Cont - 0.100*	27.7	3.1	26.8	2.9	-3.4
		Tert2	INT	42	36.15	12.27	39	36.30	14.65	0.4	0.15		28.8	2.6	33.1	2.5	14.7
			CON	53	33.42	13.57	52	31.66	12.54	-5.3			27.7	2.7	25.8	2.6	-6.9
		Tert3	INT	62	35.16	13.04	34	34.74	15.40	-1.2	0.27		28.7	3.1	28.8	3.3	0.4
			CON	30	33.43	13.72	20	29.39	9.71	-12.1			31.4	3.2	26.6	3.5	-15.5
	% of Day	Tert1	INT	35	7.9	2.67	19	8.79	3.31	11.3	0.48	*Tert - 0.015*	5.97	0.77	8.64	0.77	44.7
			CON	56	8.37	2.49	49	8.03	2.43	-4.1		*Cont - 0.082*	6.06	0.74	6.00	0.70	-1.0
		Tert2	INT	42	8.53	2.96	39	8.66	3.26	1.5	0.14		6.49	0.62	7.53	0.62	15.9
			CON	53	7.89	2.78	52	7.62	2.95	-3.4			6.10	0.65	5.75	0.63	-5.7
		Tert3	INT	62	8.32	2.87	34	8.06	3.32	-3.1	0.28		6.38	0.74	6.44	0.80	0.8
			CON	30	8.33	3.17	20	7.23	2.34	-13.2			7.09	0.78	5.95	0.85	-16.0
Sedentary Time	Minutes per Day	Tert1	INT	35	286.21	46.79	19	253.26	33.48	-11.5	-1.02	*Tert - 0.005*	300.6	7.8	273.2	7.8	-9.1
			CON	56	273.28	35.82	49	282.36	41.38	3.3		*Cont - 0.023*	297.8	7.6	298.8	7.1	0.3
		Tert2	INT	42	275.53	35.16	39	266.6	35.63	-3.2	-0.41		291.3	6.3	277.5	6.2	-4.7
			CON	53	271.63	33.41	52	276.79	41.2	1.9			293.7	6.6	299.0	6.3	1.8
		Tert3	INT	62	267.81	37.2	34	278.8	36.89	4.1	-0.01		287.5	7.5	290.7	8.0	1.1
			CON	30	261.34	35.14	20	272.65	34.02	4.3			286.4	7.9	290.2	8.6	1.3
	% of Day	Tert1	INT	35	66.1	6.56	19	64.26	7.79	-2.8	-0.40	*Tert - 0.009*	72.7	1.9	66.4	1.9	-8.7
			CON	56	64.84	6.67	49	65.62	7.67	1.2		*Cont - 0.033*	73.3	1.9	72.9	1.8	-0.5
		Tert2	INT	42	65.17	5.9	39	64.4	7.78	-1.2	-0.28		71.2	1.6	67.8	1.5	-4.7
			CON	53	65.82	7.75	52	66.93	6.93	1.7			72.3	1.6	73.0	1.6	1.0
		Tert3	INT	62	64.22	8.02	34	65.56	8.24	2.1	0.14		70.7	1.8	71.2	2.0	0.7
			CON	30	66.53	6.91	20	66.81	6.44	0.4			70.1	1.9	70.7	2.1	0.8

AGE x GROUP x TIME interaction tested using age at baseline as continuous (Cont.) and tertile (tert) level variable.
Age at Tert 1 (M = 3.3 SD = 0.21), Tert 2 (M = 3.8 SD = 0.13), Tert 3 (M = 4.5 SD = 0.34)

Age of children in each tertile						
Age Group	N	Mean	STD	Mediar	MIN	MAX
Tert1	91	3.32	0.21	3.37	2.64	3.59
Tert2	95	3.83	0.13	3.84	3.60	4.05
Tert3	92	4.49	0.34	4.39	4.05	5.44
